# Pharmaceutical policies in a crisis? Challenges and solutions identified at the PPRI Conference

**DOI:** 10.1186/s40545-016-0056-8

**Published:** 2016-03-15

**Authors:** Sabine Vogler, Nina Zimmermann, Alessandra Ferrario, Veronika J. Wirtz, Kees de Joncheere, Hanne Bak Pedersen, Guillaume Dedet, Valérie Paris, Aukje K. Mantel-Teeuwisse, Zaheer-Ud-Din Babar, on behalf of the Scientific Programme Committee of the 2015 PPRI Conference

**Affiliations:** WHO Collaborating Centre for Pharmaceutical Pricing and Reimbursement Policies, Health Economics Department, Gesundheit Österreich GmbH (Austrian Public Health Institute), Vienna, 1010 Austria; LSE Health and Department of Social Policy, London School of Economics and Political Science, London, WC2A 2AE UK; Department of Global Health, Boston University School of Public Health, Boston, MA 02118 USA; Essential Medicines and Health Products Department (EMP), World Health Organization (WHO), 1211 Geneva 27, Switzerland; World Health Organization (WHO) Regional Office for Europe, Copenhagen, 2100 Denmark; Health Division, Organisation for Economic Co-operation and Development (OECD), Paris, 75116 France; WHO Collaborating Centre for Pharmaceutical Policy and Regulation, Utrecht Institute for Pharmaceutical Sciences (UIPS), Utrecht University, Utrecht, 3508 TB The Netherlands; School of Pharmacy, Faculty of Medical and Health Sciences, University of Auckland, Private Mail Bag 92019, Auckland, New Zealand

**Keywords:** Pricing, Reimbursement, Medicine prices, High-cost medicines, Biosimilars, Europe, Challenges, Debate, Cooperation

## Abstract

In October 2015, the third international Pharmaceutical Pricing and Reimbursement Information (PPRI) Conference was held in Vienna to foster discussion on challenges in pricing and reimbursement policies for medicines. The research presented highlighted that commonly used pharmaceutical pricing and reimbursement policies are not sufficiently effective to address current challenges. Conference participants called for fundamental reforms to ensure access to medicines, particularly to new and potentially more effective and/or safe medicines, while safeguarding the financial sustainability of health systems and working towards universal health coverage.

The Vienna WHO Collaborating Centre for Pharmaceutical Pricing and Reimbursement held its third international Pharmaceutical Pricing and Reimbursement Information (PPRI) Conference in Vienna on 12 and 13 October 2015, coinciding with the tenth anniversary of PPRI, a network of competent authorities for pharmaceutical pricing and reimbursement from 46 countries [1].

The conference aimed to present the latest pharmaceutical policy analyses and to foster discussion on challenges in pricing and reimbursement policies.

The Conference had a special focus on challenges beyond the financial crisis as some countries had been hit hard by the global financial crisis, and many more have been struggling to ensure access to medicines for their population. In addition to funding cuts due to the recession, public health care systems have been struggling with the entry of a number of new high-priced medicines in the past years. The conference addressed this topic through presentations and debates in plenary as well as in the three parallel sessions. The latter related to ‘Challenges and Opportunities for Pricing and Reimbursement Policies’ [2], ‘Policies beyond the Crisis: Lessons Learned’ [3], and ‘Policy Cooperation and Interface Issues’ [4].

Around 260 people from 56 countries attended the conference. Sixty conference delegates contributed to the event as speakers, panellists or facilitators. High-level representatives of the World Health Organization (WHO), competent authorities and associations as well as outstanding researchers gave key-note speeches and made interventions during debates. Abstracts submitted by researchers had been evaluated and ranked by the Scientific Programme Committee. Authors of high-ranking abstracts were invited for oral or poster presentation. Kim Pauwels (KU Leuven, Belgium) received the Young Researcher Award; Susanne Spillane (National Centre for Pharmacoeconomics, Ireland) and Jaana Martikainen (Social Insurance Institution, Finland) were awarded for the best oral presentation and the best poster, respectively.

## High-priced medicines

From the very beginning of the PPRI Conference, which started with the key-note speech of Suzanne Hill, Senior Adviser at the Essential Medicines and Health Products Department of WHO and a stakeholder roundtable (representatives from the payers, regulatory authorities, industry and consumers), conference participants pointed to the urgent need for pharmaceutical policy reforms. While reforms would need to respond to the impact of financial austerity, more importantly, they should address existing inefficiencies in health and pharmaceutical systems. The need for reform is becoming all the more urgent due to affordability and opportunity costs issues. These are driven by several factors including the very high prices of some new medicines - such as treatments for hepatitis C, cancer and rare conditions - the upcoming marketing of high-priced medicines currently under development, demographic and epidemiological changes, and the wide disparities in available resources across and within countries. It was acknowledged that a reimbursement decision related to one single medicine could impose a high financial burden for the whole health care system and could eventually limit or crowd out other effective medicines, technologies or health ressources.

Medicines with high budget impact have also challenged the role of economic evaluations, as Florent Dromzée of the French Ministry of Health explained in his case study on sofosbuvir. The assessment of sofosbuvir highlighted the limitations of the cost-effectiveness analysis and the importance of distinguishing efficiency and affordability. Five new direct acting oral antiretroviral medicines have recently come on the market transforming the treatment of chronic hepatitis C, and sixteen new oncology medicines were added to the WHO Essential Medicines List (EML) during the 2015 review [5]. These are very effective medicines but unaffordable for many countries, as explained Nicola Magrini, Essential Medicines List Secretary at WHO. Including such medicines in the list is seen as a first step towards increasing access for patients together with comprehensive essential medicines policies [6]. Their listing on the WHO EML is envisaged to send a strong message that these medicines need to become affordable. Some tools to achieve this are already available – differential pricing, generic competition, and joint and/or strategic procurement–, but their implementation remains often limited and/or not targeted to where need is greatest.

## Barriers to access

Limited access to medicines is no longer solely an issue for low- and middle-income countries. In the stakeholder roundtable, Tim Reed of Health Action International quoted a World Health Assembly delegate commenting on the challenges of affordability and availability of medicines at the global level by extending a ‘Welcome to our world of scarcity!’ to high-income countries. Several conference abstracts identified variations and limitations not only related to affordability but also in the availability of medicines in different countries and regions [7–9].

In addition to limited accessibility of high-priced medicines, an increasing number of countries, particularly low- and middle-income countries, face problems of availability of some ‘old’ off-patent medicines with low prices, that are no longer produced and marketed, as Suzanne Hill stressed in her key-note talk. These products are essential medicines that had been marketed for a long time but whose prices appear to not be viable or not interesting enough for industry to continue production.

## Limitations of external price referencing

While conference participants were aware of the multiple causes of availability problems related to medicines, it was acknowledged that current pricing policies are likely to be an important contributor to this problem. This is in particular true for the policy of external price referencing (EPR), i.e. the practice of using the price(s) of a medicine in one or several countries in order to derive a benchmark or reference price for the purposes of setting or negotiating the price of the product in a given country [10]. Use of external price referencing incentivises manufacturers to first launch a medicine in countries with high prices and to delay market entry or not launch at all in lower-income countries in order not to reduce the benchmark reference price. This issue was addressed in several discussions at the PPRI Conference.

The limitation of using list prices when conducting EPR instead of actual discounted prices was also highlighted during the Conference. Payers are aware that with this policy they are likely to over-pay. Threats by industry that price transparency will prevent them from continuing to offer discounts to individual countries does not incentivise many countries to share data although the case of hepatitis C seems to have started triggering a change in attitude. Several conference delegates, in particular consumers and patients, called for more transparency about which elements go into the final medicine prices, and for a disclosure of discounts. Peter Schneider of the Austrian Public Health Institute illustrated, in an exploratory case study, how the results of a European price comparison would change, when the published discounted prices in Germany were used instead of list prices (9).

In many European countries external price referencing remains a key pricing policy for new medicines, as the country presentations and posters demonstrated [11].

## Are managed-entry agreements the answer?

A survey among competent authorities in Europe, undertaken in the framework of a technical review of WHO Europe on access to new medicines [12], confirmed, in principle, the use of ‘traditional’ policies for new medicines. However, pharmaco-economic evaluations and health technology assessments (HTA) as well as managed-entry agreements (MEAs) play an increasing role for new high-priced medicines [13].

Suggestions were made on how to make better use of HTA and pharmaco-economic evaluations in the decision-making process: Aris Angelis (London School of Economics, UK) proposed the application of multi-decision criteria analysis methods in reimbursement decisions [14, 15]. Based on the example of national assessments for sofosbuvir analysed in the framework of the EUNetHTA project, Wim Goettsch (National Health Care Institute, the Netherlands) urged for improved cooperation on HTA in Europe. Seven months after sofosbuvir received market authorisation, the assessment of the effectiveness had not yet started in 11 countries (thereof five European countries where no application for reimbursement had been submitted), it was ongoing in nine and it was completed in eight. Wim Goettsch concluded that marketing authorization holders appeared to set the pace of HTA assessments. This reduced the possibilities for payers to participate in voluntary joint price negotiations [16].

Managed-entry agreements were proposed as a possible solution, at least short-term, to grant access to new medicines that seemed to be providing therapeutic benefits for patients. But their limitations were clearly addressed by several conference participants. For example, Suzanne Hill from WHO reminded payers that opting for MEAs may mean implicitly accepting high (list) prices. In her presentation on high-cost cancer medicines in Australia, Agnes Vitry (University of South Australia) called for more transparency related to confidential MEAs [17]. In the stakeholder roundtable, Richard Bergström of the European Federation of Pharmaceutical Industries and Associations (EFPIA) expressed the industry’s preference for MEAs. He is convinced that confidential discounts, such as those connected to MEAs, would remain in future. However, he challenged payers for not yielding better negotiation results: ‘Despite being monopsonists, you are not good purchasers.’ The different perspectives with regard to MEAs were also described in a qualitative research performed by Kim Pauwels (KU Leuven, Belgium). Her study showed the advantages and disadvantages of MEAs as perceived by different stakeholders in Belgium and suggested a lack of trust of payers in pharmaceutical companies and vice versa [18].

## Be prepared!

The watchword of the PPRI conference was: ‘Be prepared!’ Conference delegates stressed the relevance of horizon scanning exercises, i.e. identification and early assessments of new and emerging technologies in healthcare to support decision-making, and of the authorities’ cooperation with pharmaceutical industry to obtain information on new medicines under development. There was agreement that horizon scanning is not an easy task since this requires a body of data that might not be easily accessible. Industry noted that, for the time being, it appears to be misaligned with other activities undertaken by policy-makers. Anna Nachtnebel (Ludwig Boltzmann Institute for Health Technology Assessment, Austria) reported on the strengths and weaknesses of early awareness and alert systems in the area of oncology medicines to inform authorities about medicines technologies that may have a significant impact on the health care system [19].

## Generic and biosimilar competition

Although a major part of the discussion was focused on new, high-priced medicines, generics and biosimilar medicines were also addressed as a possible part of the solution in managing pharmaceutical expenditure and improving access to medicines in European countries and globally. While generics are expected to drive medicine prices down, it was stressed how important the design of policy measures is in order to reap the benefits of generics competition. Examples from Belgium and Finland were presented where the reference price systems (internal price referencing based on generics) did not prove to be as successful as expected due to their policy design [20, 21].

In his key-note speech, Arnold Vulto (Erasmus University Medical Center Rotterdam, the Netherlands) showed the potential of biosimilar medicines in lowering prices, but he had some warnings: the uptake of biosimilar medicines might be slower than that of generics and will rely on the creation of trust and confidence among all the stakeholders involved such as prescribers, pharmacists, patients. In addition, while the cost per treatment day would likely go down, the overall public expenditure could grow due to increased access.

## Conclusions

The PPRI Conference concluded that, in order to address the outlined challenges, it is important to use a mix of policies to address the numerous and complex issues affecting access to on-patent and off-patent medicines. In line with the WHO Review on Access to New Medicines in Europe [12] it was suggested that - in addition to common and new approaches in pricing and reimbursement (peri-launch activities) - policy-makers should consider applying the full spectrum of policy options, including pre-launch activities that provide a forward-looking perspective on new medicines in development and post-launch activities that address responsible and sustainable use of medicines.

Moreover, a broader review beyond pricing and reimbursement policies is needed that includes the current patent system and considers new models that aim to de-link a medicine price from the return on investment into research and development [22, 23]. Several conference presenters and participants called for more transparency related to the costs of R&D.

Any discussion on new strategies requires dialogue with all relevant stakeholders. The role of patients and citizens was stressed during the PPRI conference whose closing commentary entitled ‘Patients have the last word?’ was given by Nicola Bedlington of the European Patients Forum. She explained that patients can understand and are willing to accept priority setting by policy-makers if the decisions were appropriately justified and communicated.

The PPRI Conference identified areas of particular relevance for collaborative approaches among policy-makers and stakeholders. Cooperation related to HTA and horizon scanning should continue and be extended. Furthermore, participants recommended joining forces on strategic procurement and on measures beyond pricing policies.

## Further information

All materials of the conference (presentations, abstract poster book, country poster book) are freely available for download at the PPRI Conference website: http://whocc.goeg.at/Conference2015/Programme.

The accepted abstracts were, together with editorials and commentaries, published in the PPRI Conference Supplement: http://www.joppp.org/supplements/8/S1/all.
